# Enhanced longevity and metabolism by brown adipose tissue with disruption of the regulator of G protein signaling 14

**DOI:** 10.1111/acel.12751

**Published:** 2018-04-14

**Authors:** Dorothy E. Vatner, Jie Zhang, Marko Oydanich, John Guers, Elena Katsyuba, Lin Yan, David Sinclair, Johan Auwerx, Stephen F. Vatner

**Affiliations:** ^1^ Department of Cell Biology & Molecular Medicine Rutgers University‐New Jersey Medical School Newark NJ USA; ^2^ Laboratory of Integrative and Systems Physiology Ecole Polytechnique Fédérale de Lausanne (EPFL) Lausanne Switzerland; ^3^ Department of Genetics Harvard Medical School Boston MA USA

**Keywords:** brown adipose tissue, G proteins, longevity, obesity

## Abstract

Disruption of the regulator for G protein signaling 14 (RGS14) knockout (KO) in mice extends their lifespan and has multiple beneficial effects related to healthful aging, that is, protection from obesity, as reflected by reduced white adipose tissue, protection against cold exposure, and improved metabolism. The observed beneficial effects were mediated by improved mitochondrial function. But most importantly, the main mechanism responsible for the salutary properties of the RGS14 KO involved an increase in brown adipose tissue (BAT), which was confirmed by surgical BAT removal and transplantation to wild‐type (WT) mice, a surgical simulation of a molecular knockout. This technique reversed the phenotype of the RGS14 KO and WT, resulting in loss of the improved metabolism and protection against cold exposure in RGS14 KO and conferring this protection to the WT BAT recipients. Another mechanism mediating the salutary features in the RGS14 KO was increased SIRT3. This mechanism was confirmed in the RGS14 X SIRT3 double KO, which no longer demonstrated improved metabolism and protection against cold exposure. Loss of function of the *Caenorhabditis elegans* RGS‐14 homolog confirmed the evolutionary conservation of this mechanism. Thus, disruption of RGS14 is a model of healthful aging, as it not only enhances lifespan, but also protects against obesity and cold exposure and improves metabolism with a key mechanism of increased BAT, which, when removed, eliminates the features of healthful aging.

## 1 INTRODUCTION

With the majority of the world's population now experiencing an increase in lifespan due to changes in lifestyle and improved therapeutic interventions, the emphasis of aging research has shifted to identify new ways to promote healthful aging. This is important because even if lifespan has increased, there are still debilitating aspects of aging that remain and those improved therapeutic interventions do not eliminate the diseases and complications, even if they reduce incidence of early death. One consequence of reducing early death is having more elderly patients, which, in turn, increases the numbers of patients with chronic diseases, such as obesity, type 2 diabetes, heart disease, neurodegeneration, and cancer (Hebert, Weuve, Scherr & Evans, 2013; NCHS 2015; NCI 2016; Prevention 2017). This exerts a severe impact on healthful lifespan, the time that people are living in good health. Accordingly, new mechanisms that not only extend lifespan but also correct some of the comorbidities of old age are of importance.

There are two distinctly different types of fat found in mammals: white adipose tissue (WAT), which is an essential site for triglyceride storage, and brown adipose tissue (BAT). The BAT is a protective mechanism of recent interest. BAT enhances energy metabolism and protects against cold exposure and obesity (Stanford et al., 2013). A novel model to investigate the role of BAT in healthful aging and lifespan is the mouse model of the disruption of the regulator for G protein signaling 14 (RGS14), which has increased BAT. RGS14 is a complex RGS family member that contains a canonical RGS domain, a tandem (R1 and R2) Ras/Rap binding domain (Kiel et al., 2005; Wohlgemuth et al., 2005), as well as a GoLoco/GPR motif. Most prior work on RGS14 focused on its effects on embryonic development and on the visual cortex and central nervous system (Evans, Lee, Smith & Hepler, 2014; Lee et al., 2010; Lopez‐Aranda et al., 2009; Shu, Ramineni & Hepler, 2010; Vellano, Brown, Blumer & Hepler, 2013). The role of BAT in RGS14 KO and its ability to enhance lifespan and improve metabolism, the focus of the present investigation, have never been explored. Sirtuins, which have been shown to be involved in mediating healthful aging (Merksamer et al., 2013; Sack & Finkel, 2012) were also examined. To confirm the essential role of BAT in mediating the protection in the RGS14 KO, we transplanted BAT from RGS14 KO to WT mice, a technique that is equivalent to a BAT KO, as it disrupts the salutary phenotype in the RGS14 KO and transplants these features to their WT, receiving the BAT. Finally, the evolutionary conservation of the effects of RGS14 loss of function (LOF) on aging was confirmed in *Caenorhabditis elegans*.

## 2 RESULTS

### 2.1 RGS14‐deficient mice exhibit extended lifespan

**Figure 1:**
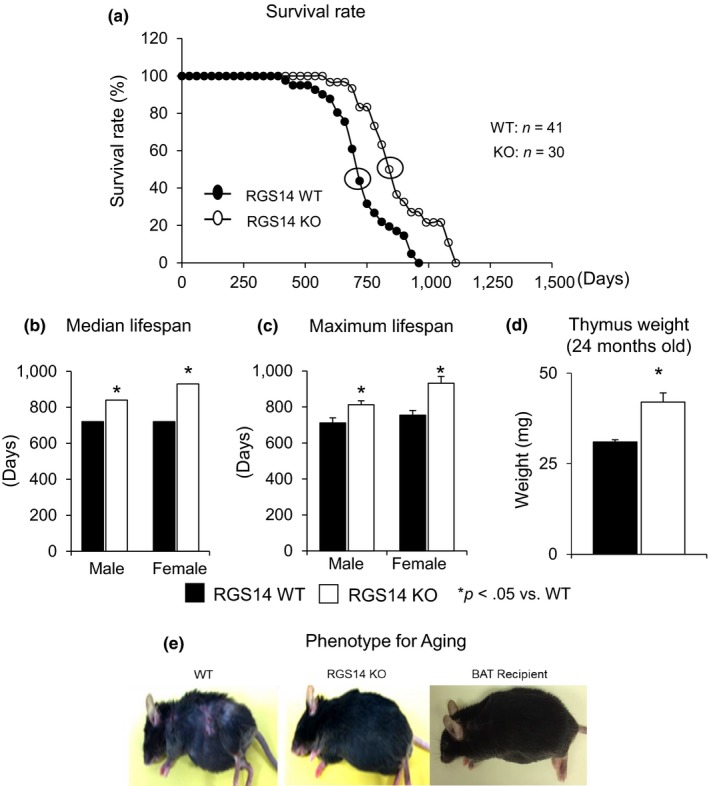
RGS14 KO model of longevity. (a) Kaplan–Meier survival curves for RGS14 KO (*n* = 30) and WT (*n* = 41) mice showed significantly augmented survival in RGS14 KO. (b, c) Median survival value and maximum lifespan were significantly greater in RGS14 KO males (*n* = 15) and females (*n* = 15) than in WT mice for both males (*n* = 20) and females (*n* = 21). (d) 24‐month‐old RGS14 KO mice showed less atrophy of the thymus, compared to WT of the same age (*n* = 3/group). (e) Furthermore, 24‐month‐old RGS14 KO mice did not show the aging phenotype normally present in WT mice of similar age, including body atrophy, loss of hair and graying of fur color. In support of the key role of BAT in aging, old WT BAT recipient mice which had BAT transplanted at 3–4 months of age had the appearance of healthful aging similar to the old RGS14 KO mice. A representative example of each is shown in panel D. For median lifespan analysis, a Mood's median test was used to determine differences in median lifespan. A Student's *t* test was used to test differences in maximum lifespan. *p < .05 vs. WT

The lifespan was monitored in the mice, and a Kaplan–Meier survival analysis demonstrated significantly longer lifespan of RGS14 KO vs. WT mice (*p* < .05). Mood's median test demonstrated that median lifespan was increased by 4 months from 24 to 28 months (*p* < .05) (Figure 1a). Median lifespan and maximum lifespan were increased to a similar extent in females and males, *p* < .05 (Figure 1b,c). The older RGS14 KO mice were also protected from aging‐induced atrophy of the thymus (Figure 1d). It is also important that BAT protects against the aging phenotype, for example, graying and loss of hair, dermatitis, and hunched back, all of which were observed in old WT mice, but not observed in old RGS14 KO mice or in old WT mice, which received BAT transplants. Representative examples are shown in Figure 1e. All of the above support the role of disruption of RGS14 and BAT in healthful aging.

### 2.2 RGS14 deficiency improves body composition and metabolism

**Figure 2:**
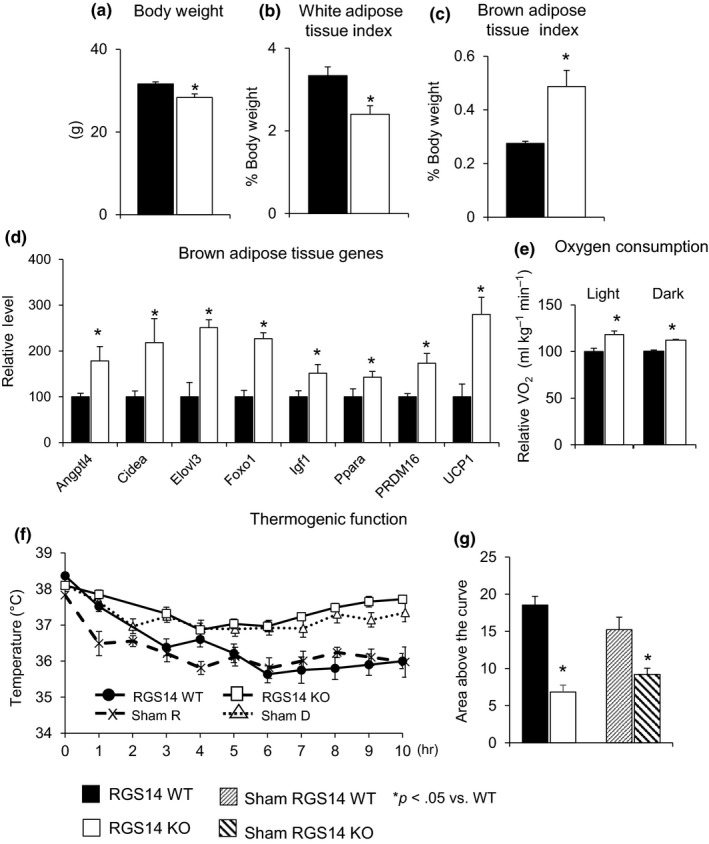
Body composition and metabolism in RGS14 KO. (a) Body weight (BW) and (b) white adipose tissue index were reduced, and (c) brown adipose tissue index was increased in RGS14 KO compared with WT (*n* = 6 in RGS14 KO; *n* = 8 in RGS14 WT). (d) Expression of BAT‐specific genes was increased in RGS14 KO BAT. (e) RGS14 KO mice had an increased VO_2_ in both the dark and the light cycles (*n* = 6/group). (f, g) RGS14 KO mice were able to keep their body temperature higher than WT animals (*n* = 6/group) when exposed to 4°C. The mock surgeries, to simulate BAT transplant surgeries, but without removing BAT, did not change the thermogenic function protection in RGS14 KO. Results are expressed as mean ± *SEM*. Statistical significance was determined by the use of a Student's *t* test or one‐way ANOVA with a Bonferroni analysis. *p < .05 vs. WT

RGS14 KO mice had improved body composition compared to WT mice. RGS14 KO mice had lower body weight (Figure 2a) and WAT index (% of white fat to total body weight) (Figure 2b). The brown fat index (% of brown fat to total body weight) was increased in RGS14 KO by 77% (*n* = 6) compared to their WT littermates (*n* = 8) (Figure 2c). From RT–qPCR analysis to profile changes in BAT transcript levels, we found that BAT‐specific markers were significantly upregulated (Figure 2d). As healthful longevity and BAT are known to improve metabolic function, we assessed metabolism through indirect calorimetry and demonstrated greater oxygen consumption in RGS14 KO (*n* = 6) than WT mice (*n* = 6) in both the light and the dark cycles (Figure 2e).

As BAT is known to protect animals from the cold (Stanford et al., 2013), we induced a cold challenge and found that body temperature was preserved better in RGS14 KO than in WT littermates (Figure 2f,g), with basal body temperatures similar in both the dark and the light cycles. In addition, mock surgery of BAT transplant in RGS14 KO and its WT littermates, that is, conducting the surgical procedure, but not removing BAT from RGS14 KO, did not alter the pattern of thermogenic function protection in RGS14 KO (Figure 2f,g). These data, taken together with Figure 1, demonstrate an increased BAT mass, improved metabolism, and protection against obesity and cold exposure in RGS14 KO mice.

### 2.3 RGS14 KO improves metabolism and mitochondrial function

**Figure 3:**
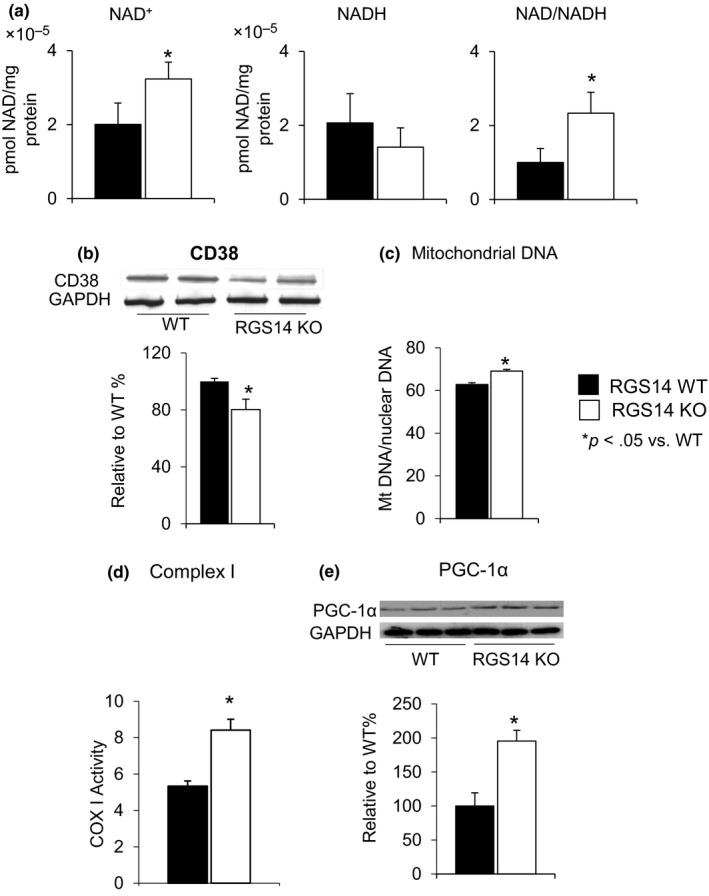
Increased NAD^+^/NADH ratio in RGS14 KO in skeletal muscle. (a) Both the absolute level of NAD^+^ and NAD^+^/NADH ratio were elevated in the RGS14 KO mice (*n* = 4/group). (b) CD38, a mechanism for changes in NAD^+^, was reduced in RGS14 KO skeletal muscle, further confirmed in the increased NAD^+^ level (*n* = 6–7/group). (c) Increase in mitochondrial DNA content in skeletal muscle was measured by mitochondrial DNA/nuclear DNA ratio (*n* = 5/group). (d) Complex I activity in skeletal muscle was significantly increased in RGS14 KO animals (*n* = 7/group). (e) PGC‐1α, a key regulator of mitochondrial biogenesis, was increased at the protein level in the BAT of the RGS14 KO mice (*n* = 3/group). Results are expressed as mean ± *SEM*. Statistical significance was determined by Student's *t* test *p < .05 vs. WT

Both the absolute levels of NAD^+^ and ratio of NAD^+^/NADH were elevated in skeletal muscle in the RGS14 KO mice (*n* = 4) (Figure 3a). To determine the mechanism and signaling pathways accounting for the induction of NAD^+^/NADH levels and SIRT3 by RGS14 deficiency, we investigated the CD38 level in skeletal muscle, which is the primary NADase in mammals and regulates cellular levels of NAD (Aksoy, White, Thompson & Chini, 2006; Camacho‐Pereira et al., 2016; Young, Choleris, Lund & Kirkland, 2006). Decreased CD38 level in RGS14 KO (Figure 3b) was found (*n* = 6–7/group), supporting the increased NAD^+^ level in Figure 3a. In addition, the RGS14 KO mice displayed increased mitochondrial content in skeletal muscle, as quantified by mitochondrial DNA (mDNA) over nuclear DNA (nDNA) ratio (Figure 3c) (*n* = 5/group). Complex I activity in skeletal muscle of the RGS14 KO mice was also significantly increased (Figure 3d) (*n* = 7/group). Expression of PGC‐1α (Figure 3e), a key regulator of mitochondrial biogenesis, was increased in the skeletal muscle of RGS14 KO animals.

### 2.4 Thermogenic and mitochondrial functions were protected by increased SIRT3 in RGS14 KO

**Figure 4:**
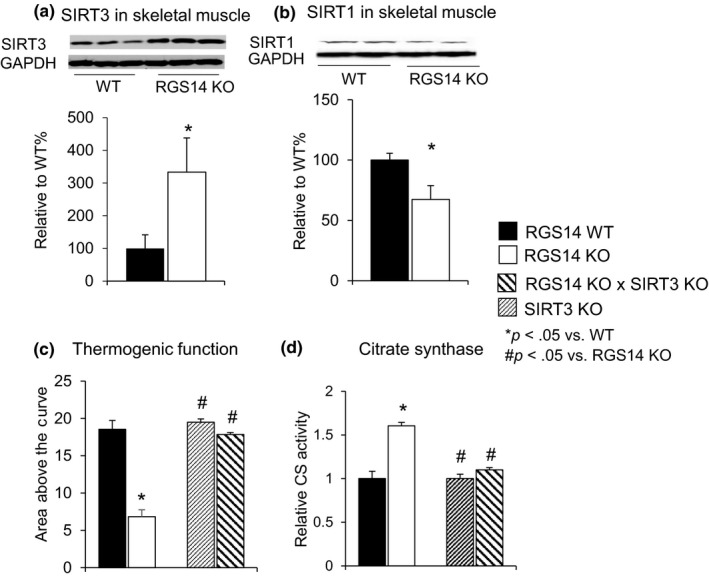
RGS14 KO have increased SIRT3, but not SIRT1. (a) SIRT3 protein levels were increased in skeletal muscle of RGS14 KO mice (*n* = 5/group), (b) SIRT1 protein levels were decreased in skeletal muscle of RGS14 KO mice (*n* = 5/group). (c) The SIRT3 KO did not show thermogenic protection, and when combined with the RGS14 KO in the RGS14 × SIRT3 double KO, the thermogenic protection was no longer present in the RGS14 KO mice (*n* = 6/group) when exposed to 4°C. (d) The increase in citrate synthase activity in skeletal muscle in RGS14 KO was also abolished in the RGS14 × SIRT3 double KO (*n* = 5/group). Results are expressed as mean ± *SEM*. Statistical significance was determined by the use of a Student's *t* test or one‐way ANOVA with a Bonferroni analysis. *p < .05 vs. WT, #*p* < .05 vs. RGS14 KO

As SIRT3 is related to WAT browning (Rouble & Storey, 2015), we assessed the expression of SIRT3 through Western blotting and found that SIRT3 protein expression was elevated robustly in skeletal muscles of the RGS14 KO mice (Figure 4a), whereas SIRT1 was actually decreased (Figure 4b). SIRT3 also improved thermogenesis and mitochondrial biogenesis, and other important mechanisms mediating healthful aging. The essential role of SIRT3 was confirmed by loss of thermogenic protection in the RGS14 x SIRT3 double KO (Figure 4c) (*n* = 5) and loss of increased citrate synthase activity in the RGS14 X SIRT3 double KO mice (*n* = 5) (Figure 4d).

### 2.5 Healthful aging in RGS14 KO mice is mediated by BAT

**Figure 5:**
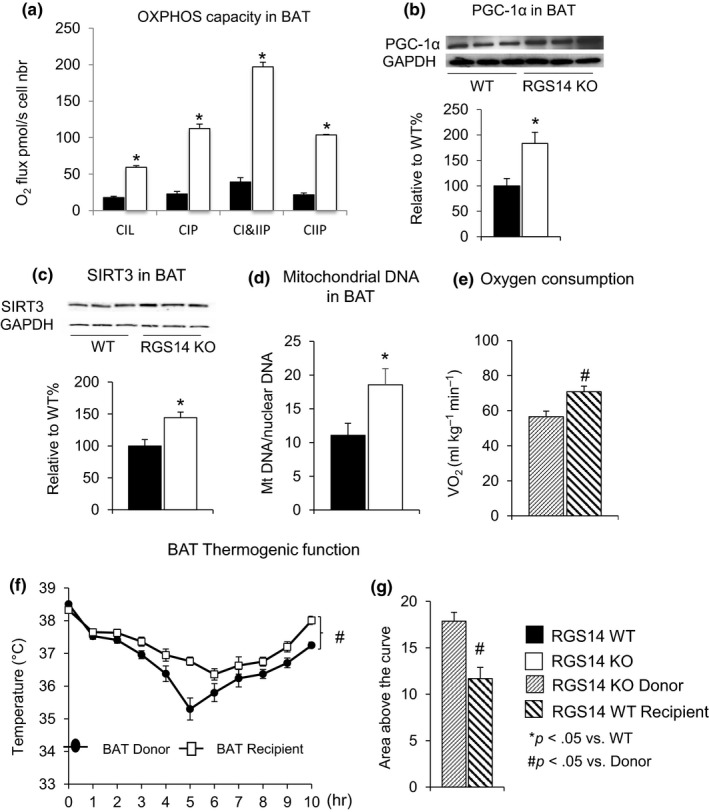
BAT mediates energy metabolism. (a) Oxidative phosphorylation (OXPHOS) capacity was increased in BAT from RGS14 KO vs. BAT from WT as reflected by increased CIL: Complex I leak respiration; CIP: OXPHOS capacity of Complex I; CI&CIIP: OXPHOS capacity of Complex I&II; and CIP: OXPHOS capacity of Complex II. (b) PGC‐1α, a key regulator of mitochondrial biogenesis, was increased at the protein level in the BAT of the RGS14 KO mice (*n* = 3/group). (c) SIRT3 protein levels were increased in BAT of RGS14 KO mice (*n* = 3/group). (d) Increased mitochondrial DNA/nuclear DNA ratio was found in BAT of RGS14 KO (*n* = 5/group). The data comparing BAT transplants and recipients are shown and demonstrate that WT BAT recipients gained the protective features of RGS14 KO and RGS14 KO lost these protective features (e–g). (e) Oxygen consumption was increased in BAT recipients compared to BAT donors (*n* = 4/group). (f) Thermogenic protection was lost in BAT donors and gained in BAT recipients (*n* = 10/group). (g) The area above the curve was significantly greater in BAT recipients than in BAT donors. Results are expressed as mean ± *SEM*. Statistical significance was determined by the use of a Student's *t* test. *p < .05 vs. WT, and #*p* < .05 vs. donor

In addition to the improved mitochondrial function in skeletal muscle, respirometry on fresh BAT from RGS14 WT and RGS14 KO was assessed, demonstrating increased oxidative phosphorylation capacity of mitochondrial complexes in RGS14 KO BAT (Figure 5a). Expression of PGC‐1α (Figure 5b), SIRT3 (Figure 5c), and mitochondrial DNA/nuclear DNA ratio (Figure 5d) was also increased in the BAT of RGS14 KO animals.

To confirm that BAT is responsible for the healthful aging in RGS14 KO mice, we transplanted BAT from the RGS14 KO mice to WT. The surgical removal of BAT is a technique that is equivalent to a BAT KO, with the hypothesis that if BAT mediated healthful aging, then the WT animals would acquire the phenotype of RGS14 KO mice and vice versa. BAT transplanted from RGS14 KO mice enhanced oxygen consumption (Figure 5e) and thermogenic protection (Figure 5f,g), mimicking the characteristics of RGS14 KO (Figure 2e–g). These effects were lost in RS14 KO donors, but gained in WT BAT recipient mice, thus further supporting the importance of BAT in mediating the increased metabolism and thermogenic protection.

### 2.6 RGS‐2 loss of function reduces oxidative stress and extends *Caenorhabditis elegans* lifespan

**Figure 6:**
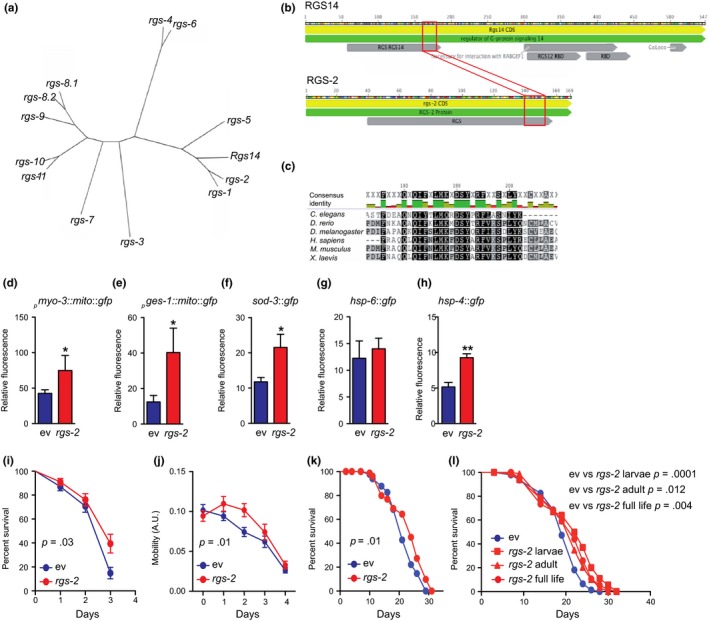
Evolutionary conservation of the RGS14 KO model of longevity. (a) Phylogenetic tree made by blasting the RGS14 amino acid sequence with all the RGS family members in the nematode *Caenorhabditis elegans*. (b, c) Alignment of RGS‐2 with the murine RGS14 (b) and other species (c) shows that the RGS domain is evolutionary conserved. (d) Knockdown of rgs‐2 by RNAi increased mitochondrial content in muscle (pmyo‐3::mito::gfp). (e) Knockdown of rgs‐2 by RNAi increased mitochondrial content in intestinal (pges‐1::mito::gfp) cells. (f) Antioxidant defense (sod‐3) is increased upon rgs‐2 RNAi. (g) Mitochondrial unfolded protein response (hsp‐6) was not affected by rgs‐2 RNAi treatment. (h) ER stress response was induced in worms fed with rgs‐2 RNAi. (d–h) Values are mean ± *SEM*,* n* = 4, where each *n* represents a pool of 20 worms. Statistical significance was determined by the use of a Student's *t* test: *p < .05, **p < .01. (i) Worm survival is preserved upon paraquat treatment (4 mM) after rgs‐2 RNAi compared to worms fed with empty vector. (j) Worm mobility is improved during aging after rgs‐2 RNAi compared to worms fed with empty vector. (k) Worms exposed to rgs‐2 RNAi live longer compared to control worms under basal conditions. (l) The observed increase in lifespan upon rgs‐2 RNAi treatment is more important when worms are exposed to rgs‐2 RNAi during larval stages than during adult life. (i–l) *n* = 100. Statistical significance was determined by the use of the log‐rank (Mantel–Cox) method. *p* < .05 is considered as significant

The nematode *C. elegans* possesses 12 RGS proteins (Sierra et al., 2002). According to the results of Basic Local Alignment Search Tool (BLAST), two members of the family, *rgs‐1* and *rgs‐2*, are closely related to the mammalian RGS14 (Figure 6a). Phylogenetic studies show that the RGS domain is evolutionarily conserved among different species, including nematode, fruit fly, zebra fish, frog, mouse, and human (Figure 6b,c).

We next explored the effects of *rgs‐2* loss of function (LOF) in *C. elegans*. By using reporter strains, expressing green fluorescent protein (GFP) in mitochondria of the body wall muscles and intestine, we were able to assess mitochondrial content in these tissues. Similar to an increase in mitochondrial number observed in RGS14 KO mice, *rgs‐2* knockdown by RNAi feeding raised mitochondrial content in worms (Figure 6d,e). By using a *sod‐3*::gfp reporter strain, we assessed *rgs‐2* RNAi effects on the oxidative stress defense activation. Worms fed with *rgs‐2* RNAi showed an induced SOD‐3 (mitochondrial SOD) expression (Figure 6f). To explore whether any other kind of stress response is involved in the *rgs‐2* LOF molecular mechanism, we looked at the mitochondrial (mt) and endoplasmic reticulum (ER) unfolded protein responses (UPR) activation. Interestingly, no UPR^mt^ involvement was detected (Figure 6g); however, there was a strong induction of the UPR^ER^ upon *rgs‐2* downregulation (Figure 6h).

Using paraquat as a well‐known reactive oxygen species (ROS) inducer, we further explored the effects of *rgs‐2* LOF on the activation of the oxidative stress defense activation system. In line with the observed increase in SOD‐3 expression, we established that worms fed with *rgs‐2* RNAi survived better with paraquat compared to worms fed with the control RNAi (Figure 6i). Importantly, at the basal condition, *rgs‐2* LOF has also shown positive effects on *C. elegans*: Similar to RGS14 KO mice, *rgs‐2* RNAi improved the worm's movement capacity (Figure 6j) and extended their lifespan (Figure 6k). By exposing worms to the *rgs‐2* RNAi at different periods of their life, we observed that the strongest lifespan extension occurs when worms are fed with *rgs‐2* RNAi during the larval stage (Figure 6l).

## 3 DISCUSSION

The major finding of this investigation was that disruption of the regulator for G protein signaling 14 is a novel model of longevity. Moreover, the RGS14 KO homolog in *C. elegans* also showed increased survival, confirming the importance of this mechanism and its evolutionary conservation. Another major finding was that the RGS14 KO is a model of healthful aging, as it also reduces WAT and improves metabolism and thermogenic function. Potentially, the most important new information is that the mechanism of the healthful aging in the RGS14 KO involves BAT, which is increased in this model. In support of the importance of BAT in healthful aging, BAT mass and BAT function are known to decay with aging (McDonald & Horwitz, 1999), while WAT mass is known to increase (Goncalves et al., 2017). Indeed, we found that the signature improvements in metabolism observed in the skeletal muscle of the RGS14 KO were recapitulated in BAT from RGS14 KO. These include enhanced oxidative phosphorylation, PGC‐1α, SIRT3, and mitochondrial DNA (Figure 5).

Another major finding of the current investigation was that the RGS14 KO mice showed an extension in lifespan in both males and females and did not exhibit many of the adverse effects of aging normally observed in mice, such as decreased thymus weight, body atrophy, or loss of body hair (Gui, Mustachio, Su & Craig, 2012; Tyner et al., 2002). The current field of aging‐related research is focused more on extending a healthful lifespan, rather than simply extending lifespan, as older individuals with complications of aging that impair the quality of life often do not feel that further extension of lifespan is desirable. It was therefore important to determine whether these mice were also protected from risk factors that impair healthful aging, for example, protection against obesity (American Diabetes, 2004; Brown, Fujioka, Wilson & Woodworth, 2009; Guh et al., 2009) and impaired metabolism (Finkel, 2015; Nguyen, Samson, Reddy, Gonzalez & Sekhar, 2013; Roberts & Rosenberg, 2006). Indeed, the RGS14 KO mice have a reduced WAT/body weight ratio, are protected against the cold, and have increased mitochondrial biogenesis.

To prove that the increase in BAT activity was key for the improved metabolism and healthful aging in the RGS14 KO mice, we utilized a BAT KO simulation, that is, surgically removing BAT from RGS14 KO mice and transplanting it into WT recipients. The BAT transplantation reversed the phenotype of the RGS14 KO and WT; that is, WT BAT recipients had improved oxygen consumption and protection from cold exposure, thus demonstrating a phenotype similar to that of RGS14 KO with intact BAT, whereas the RGS14 KO donors lost these salutary features and were no longer different from WT. Thus, the WT mice with RGS14 KO BAT transplantation did not show the adverse aging‐related features observed in WT mice without the BAT transplants. All of the above point to BAT as a key mechanism mediating the enhanced metabolism and healthful aging in the RGS14 KO mouse model.

Most prior studies have found that increased SIRT1 is involved in healthful aging (Herranz et al., 2010; Sasaki et al., 2014; Satoh et al., 2013), whereas the results for SIRT3 are more controversial (Bellizzi et al., 2005; De Rango et al., 2008; Lescai et al., 2009; McDonnell, Peterson, Bomze & Hirschey, 2015; Rose et al., 2003). However, decreases in SIRT1 have been associated with cancer protection, which also is involved in healthful aging (Chen et al., 2014; Ohanna et al., 2014). In the RGS14 KO, SIRT1 was downregulated, while SIRT3 was upregulated. To confirm the role of the SIRT3 mechanism, a double KO (RGS14 KO X SIRT3 KO) was studied. The RGS14 X SIRT3 double KO mice lost their improved metabolism and thermogenic protection, pointing to SIRT3 as a mediator of the beneficial effects on metabolic regulation in the RGS14 KO animals. Therefore, RGS14 deficiency promotes increased SIRT3 activity, not only by increasing its expression levels, but also by increasing the availability of NAD^+^, an important cofactor required for sirtuin function. SIRT3 activation, in turn, leads to improved mitochondrial biogenesis, providing the molecular basis for healthful aging in the RGS14 KO animals.

In summary, increased BAT in the RGS14 KO constitutes a novel model of healthful longevity. The salutary healthful aging properties of the RGS14 KO are mediated by BAT, involving a SIRT3 mechanism and its ability to improve metabolism and mitochondrial function, also known to mediate healthful aging (Bratic & Trifunovic, 2010; Brewer, Gibbs & Smith, 2016; Herranz et al., 2010). The latter point was confirmed by experiments with BAT transplants, which reversed the phenotypes of the RGS14 KO and WT, such that the WT with the BAT transplant did not exhibit the adverse phenotype of aging and demonstrated improved cold exposure tolerance and oxygen consumption, whereas the RGS14 KO donors no longer possessed these features of healthful aging. Thus, the results of this investigation support the concept that finding a mechanism to translate the RGS14 KO or BAT phenotype to patients would be a novel therapeutic approach to promote healthful longevity.

## 4 EXPERIMENTAL PROCEDURES

### 4.1 Generation of RGS14 KO mice

C57Bl6/J background mice with systemic RGS14 gene deficiency were developed as previously described (Lee et al., 2010). RGS14 KO mice (RGS14tm1‐lex) were generated in the Transgenic Core Facility at Rutgers University—New Jersey Medical School, through the National Institutes of Health‐sponsored Mutant Mouse Regional Resource Center at http://www.informatics.jax.org/searches/accession_report.cgi?id=MGI:3528963. Embryos were implanted into C57/BL6 females, and founder mice were crossed with C57/BL6 to establish RGS14‐KO. All mice were from F1 heterozygote crosses. Genotypes were determined by PCR of genomic DNA from mice tails. WT forward primer was 5′ cagcgcatcgccttctatc 3′. Primer for the targeting vector was 5′ gcagcgcatcgccttctatc 3′ with a shared reverse primer (5′ agactggcagaagaattcagg 3′). RGS14 KO × SIRT3 KO mice were generated by crossing RGS14^−/−^ and SIRT3^−/−^ (provided by Dr. David Sinclair, Harvard Medical School).

### 4.2 Sample and subject selection

Sample sizes for all experiments were predetermined using power analysis and on the basis of extensive laboratory experience with these end points. Animals that survived through to the endpoint of the successful experiments were included in the analysis. These criteria were pre‐established. No animals were excluded from the results. For all molecular and physiological experiments, animals were subjected to the same surgery and grouped on the basis of their genotypes after data collection.

### 4.3 Animal experimental procedures

Pups were weaned at 28 days of age and housed individually to allow for measurement of food intake on standard diet, in a pathogen‐free facility under a 12:12 hr light/dark cycle with access to water and food ad libitum. Age‐ and sex‐matched mice were also followed as controls. Investigators were blinded until the data were collected. No randomization was used during animal handling. Thymuses were collected and weighed at defined time points. For aging phenotype, old mice were compared at an age of 24 months old and also documented for lifespan on the day they died.

### 4.4 Calculation of adiposity index

Three‐ to five‐month‐old RGS14 KO or WT mice were sacrificed and their gonadal, retroperitoneal, inguinal, and brown fat pads were isolated and weighed. The white adiposity index was calculated using total adipose depot (gonadal, retroperitoneal, inguinal) weight divided against live body weight then multiplied by 100. The brown adiposity index was calculated using brown adipose depot weight divided against live body weight then multiplied by 100.

### 4.5 Indirect calorimetry

The mice were individually housed in separate metabolic chambers (Accuscan Instruments Inc., OH) with ad libitum access to food and water. The chambers were placed into a controlled environment with regulated temperature and 12‐hr day/night cycles. Oxygen consumption and production were recorded every 10 min for 24 hr.

### 4.6 Brown adipose tissue (BAT) removal and transplantation

Three‐ to five‐month‐old male RGS14 KO or WT mice were anesthetized with pentobarbital sodium. The backs of the mice were shaved, and the mice were placed in the prone position. A 2‐cm midscapular transverse incision was made on the back of the mouse, and the BAT was freed from surrounding muscles and removed through the skin incision. Transplantation was performed by removing BAT from RGS14 KO mice. The removed BAT from donor mice was incubated in 10 ml saline at 37°C for 20–30 min. Three‐ to five‐month‐old WT recipient mice were anesthetized. 0.1 g BAT from RGS14 KO donor was transplanted into the visceral cavity of each recipient WT mouse. The graft was carefully lodged deep between folds within the endogenous epididymal fat of the recipient mice. The skin incision in the recipient mice was either closed with 6‐0 nylon sutures or with stainless steel wound clips. The animal was allowed to recover in a warm Thermocare unit. The removed BAT was used for BAT transplantation. These mice were allowed to recover for 3 days prior to the BAT thermogenic function studies. These mice were also studied older, at 24 months of age. The sham‐operated mice underwent the same procedure, except for receiving donor tissues.

### 4.7 BAT thermogenic function

RGS14 KO and WT mice that had been acclimatized to thermoneutrality (30°C) for 4–10 days were transferred to 4°C with full access to water and food (Jin et al., 2011; Vila‐Bedmar et al., 2012). Rectal temperature of the mice was measured every hour. Comparison of BAT transplanted mice from RGS14 KO vs. WT was made at 3 days after transplantation.

### 4.8 Biochemical analyses

NAD^+^/NADH was measured using EnzyChrom^™^ NAD^+^/NADH Assay Kit from BioAssay Systems.

#### 4.8.1 Immunoblotting

Proteins separated by SDS–PAGE were transferred to nitrocellulose membranes. The membranes were probed with primary antibody at 4°C overnight. The bands were visualized using chemiluminescence reagents. The linear range of detection for different proteins and band intensities were determined by densitometry. Blots were reprobed with GAPDH to equalize sample loading. The antibodies used were SIRT3 (2627, Cell Signaling, 1:1,000), PGC‐1α (ST1203, Millipore, 1:1,000), SIRT1 (D739, Cell Signaling, 1:1,000), and CD 38 (sc‐70655, Santa Cruz Biotechnology, 1:1,000).

#### 4.8.2 Mitochondrial biogenesis and function

Mitochondrial DNA content was determined by NovaQUANT™ Mouse Mitochondrial to Nuclear Ratio Kit from EMD Millipore. Complex I activity was determined using MitoProfile Rapid Microplate Assay Kit from MitoSciences. Citrate synthase activity was determined using a Citrate Synthase Assay Kit from Sigma‐Aldrich.

#### 4.8.3 Quantitative RT–PCR

Total RNA was prepared from frozen BAT tissues or cell cultures using Trizol reagent (Sigma). The mRNA of interest was reverse‐transcribed according to standard protocol. Quantitative real‐time PCR (7700 Prizm, Perkin‐Elmer/Applied Biosystems) was performed with specific primers. Results were normalized to beta‐actin.

### 4.9 *Caenorhabditis elegans* experiments

*Caenorhabditis elegans* strains were provided by the *Caenorhabditis* Genetics Center (University of Minnesota). Worms were maintained on nematode growth medium (NGM) agar plates seeded with *E. coli* OP50 bacteria at 20°C, unless stated otherwise. For the experiments, the following strains were used: Bristol N2, NL2099 (rrf‐3(pk1426)II), SJ4143 (zcIs17[ges‐1::GFP(mit)]), SJ4103 (zcIs14[myo‐3::GFP(mit)]), KN259 (huIs33[sod‐3::GFP+pRF4(rol‐6(su1006))]), SJ4005 (zcIs4[hsp‐4::GFP]), and SJ4100 (zcIs13[hsp‐6::GFP]).

Bacterial feeding RNAi experiments were carried out as described (Kamath, Martinez‐Campos, Zipperlen, Fraser & Ahringer, 2001), and the clone, which was used for the experiments, was *rgs‐2* (F16H9.1). It was purchased from GeneService, and its identity was confirmed by sequencing.

### 4.10 Lifespan assays

*Caenorhabditis elegans* lifespan assays were carried out at 20°C as described (Mouchiroud et al., 2011). Briefly, each condition included 100 worms and the scoring was performed every 2 days until all worms were dead. The reasons for censoring were the «exploded vulva» phenotype or animals that crawled off the plate. Where indicated, paraquat was added on top of the seeded agar plates. L4 stage worms were added to these plates once the paraquat solution was completely dried. For the paraquat experiments, the scoring was performed every day for the first 3–4 days.

### 4.11 Mobility assessment

The movement of worms was recorded for 90 s at l4 stage and at days 1, 2, 3, 4, and 5 of adulthood using a Nikon DS‐L2/DS‐Fi1 camera and controller setup, attached to both a computer and a standard bright‐field microscope. One hundred worms per condition were used. The movement of worms during aging was calculated by taking an integral of the speed value, which was assessed by following the worm centroids with a modified version of the Parallel Worm Tracker for MATLAB (Mouchiroud et al., 2013).

### 4.12 GFP quantification

Fluorescence intensity in worm reporter strains expressing green fluorescent protein (GFP) was quantified using Victor X4 plate reader (Perkin‐Elmer). Eighty worms at the corresponding age were used per condition. Worms were placed into M9 medium in black‐walled 96‐well plate, with 20 worms per well. Each experiment was repeated at least twice.

### 4.13 Respirometry on fresh BAT

Fresh BAT was isolated from RGS14 WT or KO male mice. Mitochondrial function in fresh BAT was evaluated using high‐resolution respirometry (Oroboros Oxygraph‐2k; Oroboros Instruments, Austria), as previously described (Pirinen et al., 2014), with minor modifications. Briefly, Complex I leak respiration was measured by adding pyruvate (9.8 mm), glutamate (20 mm), and malate (1.6 mm). This was followed by the addition of ADP (4.8 mm) to assess Complex I OXPHOS capacity. Adding succinate (9.6 mm) stimulated Complex I + Complex II‐driven coupled respiration. Finally, Complex II OXPHOS capacity was assessed by sequential addition of rotenone (0.1 mm), which inhibited Complex I activity. O_2_ flux obtained in each step of the protocol was normalized by the weight of the tissue used for the analysis.

### 4.14 Statistics

Data were presented as mean ± *SEM*. Statistical comparisons among groups (*n* ≥ 3) were calculated using two‐way ANOVA with a Bonferroni analysis. Comparisons between two groups were calculated using a two‐tailed Student's *t* test. To examine statistical significance of the longevity data, we used three statistical tests: (i) The Kaplan–Meier analysis was used for the total survival curve, specifically the log‐rank (Mantel–Cox) test, as previously described (Bland & Altman, 2004). (ii) For median lifespan analysis, a Mood's median test (Glover et al., 2016), was used to determine differences in median lifespan. (iii) A Student's *t* test was used to test differences in maximum lifespan. *p* values of < .05 were considered significant.

### 4.15 Study approval

Animals used in this study were maintained in accordance with the Guide for the Care and Use of Laboratory Animals (National Research Council, Eighth Edition 2011). These studies were approved by the Institutional Animal Care and Use Committee of Rutgers University—New Jersey Medical School.

## AUTHOR'S CONTRIBUTION

SFV, DEV, and JA designed the study; SFV, DEV, JA, LY, JZ, JG, EK, and MO conducted the study; and SFV, DEV, JA, LY, JZ, JG, EK, DS, and MO wrote the manuscript.

## CONFLICT OF INTEREST

The authors have declared that no conflict of interest exists.
